# Universal Health Insurance Coverage and the Economic Burden of Disease in Eastern China: A Pooled Cross-Sectional Analysis From the National Health Service Survey in Jiangsu Province

**DOI:** 10.3389/fpubh.2022.738146

**Published:** 2022-02-07

**Authors:** Shenping Zhou, Chenyu Zhou, Qin Yuan, Zhonghua Wang

**Affiliations:** ^1^School of Health Policy Management, Nanjing Medical University, Nanjing, China; ^2^Institute of Healthy Jiangsu Development, Nanjing Medical University, Nanjing, China; ^3^The Public Health Policy and Management Innovation Research Team, Nanjing Medical University, Nanjing, China; ^4^Center for Global Health, Nanjing Medical University, Nanjing, China

**Keywords:** universal health insurance coverage, economic burden of disease, total health expense (THE), out-of-pocket health expense (OOP), household catastrophic health expenditure (HCHE)

## Abstract

China has achieved universal social health insurance coverage, but it is unclear whether this has alleviated the economic burden of disease for individuals. This was investigated in the present study by analyzing National Health Service Survey (2008–2018) data from Jiangsu province. Ordinary least squares and binary multivariate logistic regression of pooled cross-sectional data were carried out to evaluate the effect of universal health insurance coverage and other socioeconomic factors on the economic burden of disease. Total health expenses (THE) first increased and then decreased during the survey period while out-of-pocket health expenses (OOP) decreased except for urban residents, for whom OOP increased after 2013. Household catastrophic health expenditure (HCHE) was stable between 2008 and 2013 but increased after 2013. Social health insurance had a significant positive effect on the annual THE and OOP and a negative effect on HCHE, however, universal health insurance coverage could alleviated THE and the economic burden of disease on individuals (OOP) while it was insufficient to protect against the economic risk of diseases (HCHE), with greater benefits for urban as compared to rural residents. Other socioeconomic factors including age, marital status, education, income, and health status also influenced the economic burden of disease.

## Introduction

In 2013, the World Health Organization (WHO) put forth the concept of universal health coverage, which aims to ensure that all people can access good quality health services without falling into poverty (1). Medical expenses can directly affect the economic burden of disease on individuals, which can adversely impact their health status. The economic burden of disease refers to economic losses to patients, families, or society caused by disease, disability, and premature death (2). Countries all over the world have taken various measures to alleviate this burden such as medical insurance, which is an economic risk-sharing strategy (3) that is popular because it promotes mutual aid while reducing individual burden (4). Social health insurance systems covering the whole population have become the basis for ensuring a certain standard of national health (5).

Since the Decision on Establishing the Basic Medical Insurance System for Urban Workers in 1998, China's social health insurance system has made great progress in terms of coverage rate, which increased from 21% in 2003 (6) to more than 95% in 2020 (7). The current health insurance system framework in China consists of Urban Employees' Medical Insurance, Urban Residents' Medical Insurance (URMI), and New Rural Cooperative Medical Scheme (NCMS). In January 2016, the General Office of the State Council issued the Opinions on Integrating the Basic Medical Insurance System for Urban and Rural Residents, which proposed merging URMI and NCMS into Urban and Rural Residents' Medical Insurance.

Health insurance has been shown to reduce the economic burden of disease (8–11). In general, medical insurance can promote health and economic security (12). A study conducted in India found that community-based medical insurance reduced household catastrophic health expenditure (HCHE) (13). Health insurance involves risk-sharing, which can lower the cost of medical services and thus reduce the economic burden on patients (8). However, it has also been argued that this effect is limited and that health insurance can actually increase patients' medical expenditure (economic burden) (14–17). For example, Sepehri et al. found that insurance had no significant impact on decreasing out-of-pocket spending (18). And an analysis of the medical insurance systems in Philippines also showed that it did not significantly alleviate the economic risks of disease (19).

Even with the continuous improvement of the social health insurance system, health expenditures and the economic burden of disease are increasing for the Chinese population (20). Studies on the effect of social health insurance on the economic burden of disease in China have yielded conflicting findings. One study found that health insurance reduced the cost of health services for individuals through the sharing of health expenses (9). The NCMS (21) and URMI (22) were shown to significantly reduce the medical expenses of rural residents and alleviated the medical economic burden of rural and urban residents, respectively. However, others have demonstrated that the NCMS did not decrease the actual economic burden and incidence of critical disease (23). Although social health insurance increased health service utilization among the elderly, there was no corresponding reduction in the economic burden of disease (24). It was also determined that social health insurance increased the cost of medical services, thus increasing the economic burden on patients (14, 25). Clarifying the effect of social health insurance on the economic burden of disease is important as this can affect the choice of medical treatment and individuals' health status as well as socioeconomic development as a whole in China (19).

Most studies to date on the effect of social health insurance on the economic burden of disease have always analyzed cross-sectional data or examined specific populations or insurance types (2), however, with the adjustment and change of policies, the role of health insurance will also change. Therefore, our findings may conduct a comprehensive evaluation of the policy effect in the process of universal health insurance coverage based on the whole population. In this context, we used the data from the National Health Service Surveys (NHSS) conducted in Jiangsu province from 2008 to 2018, which spans the period of China's medical reform and the introduction of universal social health insurance coverage to investigate the effect of universal social health insurance on Chinese residents' economic burden of disease based on. Specifically, we first evaluated trends in the economic burden of disease between 2008 and 2018, then estimated the associations between universal social health insurance coverage, socioeconomic factors, and the economic burden of disease by regression analysis of pooled cross-sectional data. Our results can guide government initiatives to reduce the economic burden of disease for the population but especially for vulnerable groups, and provide a reference for establishing universal medical insurance systems in other developing countries.

## Materials and Methods

### Data Source

Data were obtained from the 4th (2008), 5th (2013), and 6th (2018) NHSSs. The survey is conducted every 5 years by the Center for Health Statistics and Information of the National Health Commission. The survey sites were selected by multistage stratified random sampling and included 156 counties (cities and districts) in 31 provinces. Five towns (streets) were randomly selected in each county, two villages (neighborhood committees) were randomly selected in each town, and 60 households were randomly selected in each village for a total survey population of nearly 300,000 from 93,600 households nationwide. Jiangsu is a densely populated province in Eastern China with a population of 80.293 million, and is one of the most developed provinces in the country. Our group organized and completed the NHSS in Jiangsu province. A total of 19 counties were surveyed; from these, 7021, 10,466, and 11,550 individuals were randomly selected in 2008, 2013, and 2018, respectively, and included in our analysis. Excluding the missing data, the whole sample included 25,042 respondents over the age of 15 years.

The data in the NHSS covered detailed personal information including demographic and socioeconomic characteristics of urban and rural residents (sex, marital status, education level, household size, employment status, income level, health insurance, self-reported health status, and chronic diseases, etc). The survey also included questions on respondents' health service utilization and medical expenses.

### Variable Selection

In most studies, the economic burden of disease on individuals is defined as medical expenses (26, 27) or the proportion of household health expenditure in household disposable income (8, 28). Considering the questionnaire content and our research objectives, we selected three dependent variables as indicators of the economic burden of disease on respondents—namely, annual total health expenses (THE), annual out-of-pocket medical expenses (OOP), and HCHE. The main explanatory variables including health insurance and other socioeconomic variables were selected based on Anderson's healthcare utilization behavior model (29–31). There were predisposing variables (demographic characteristics such as age, sex, etc), enabling variables (socioeconomic characteristics such as medical insurance, income, education level, etc), and need variables (indicators related to individual health status such as self-reported health status, chronic diseases, etc). Given that the data were pooled and cross-sectional, we also examined the effect of time and the interaction effect of time and social health insurance. Detailed descriptions of the variables can be found in (Table 1).

**Table 1:** Description of variables.

	**Category**	**Indicator/survey question**
**Dependent variable**		
Total health expenditure		Sum of outpatient and inpatient expenses in the past year
Out-of-pocket health expenditure		Sum of out-of-pocket outpatient and inpatient expenses in the past year
Household catastrophic health expenditure	0, No 1, Yes	
**Independent variable**		
**Predisposing factors**		
Sex	1, Male 2, Female	Question: What is your sex?
Age (years)	1, ≤30 2, 30–45 3, 45–60 4, >60	Question: What is your year of birth?
Household number	1, ≤2 2, >2	Question: What is the size of your household?
Marital status	1, Unmarried 2, Married	Question: What is your marital status? (Widowhood and divorced both belong to “Not married”)
Education level	1, Less than secondary 2, Upper secondary/ vocational training 3, Tertiary	Question: What is your education level?
Employment status	0, Unemployed 1, Employed 2, Retired	Question: What is your employment status?
**Enabling factor**		
Social health insurance	0, No 1, Yes	Question: Do you have any social health insurance?
Financial subsidies	0, No 1, Yes	Question: Are you a Medicaid recipient or living in a poor or low-income household?
Income level	1, Lowest 2, Lower 3, Higher 4, Highest	Yearly household income divided by the number of household members; the first household member is assigned a weight of 1, with all other members assigned a weight of 0.5
Area of residence	0, Rural 1, Urban	Question: What is your registered residence type?
**Need factor**		
Depression	0, Not depressed 1, Depressed	Question: What is the extent of your depression?(The answer is “not depressed,” “mildly depressed,” “moderately depressed” and “severely depressed,” and the last three were considered depressed.)
Self-reported health status (VAS score)	1, ≤49 2, 49–70 3, 70–80 4, 80–100	Question: How would you rate your health today on a scale of 0 (worst possible health) to 100 (best possible health)?
Smoking	0, No 1, Yes	Question: Have you ever been or are you currently a smoker?
Drinking	0, No 1, Yes	Question: Do you currently drink alcohol?
Chronic diseases	0, No chronic disease 1, 1 Chronic disease 2, >1 Chronic diseases	Questions: Do you have confirmed hypertension or diabetes? Have you been diagnosed with other chronic diseases?

*VAS, Visual Analog Scale*.

### Statistical Analysis

#### Descriptive Analysis

Data were analyzed using SPSS v25.0 (SPSS Inc, Chicago, IL, USA) and Stata v13.0 (College Station, TX, USA). The significance of differences between groups was evaluated with the Pearson chi-squared test and analysis of variance. All cost data were converted to the value in 2008.

### Calculation of the Economic Burden of Disease

Indicators of the economic burden of disease included annual THE, annual OOP, and incidence of HCHE; these were calculated with the Equations 1–3 below.


(1) $$\begin{array}{rcl} THE & = & 26*\sum_{k=1}^{n}\left(A_{k}+B_{k}\right)+\sum_{k=1}^{m}\left(C_{k}\right) \end{array}$$

(2) $$\begin{array}{rcl} OOP & = & 26*\sum_{k=1}^{n}\left(a_{k}+b_{k}\right)+\sum_{k=1}^{m}\left(c_{k}\right) \end{array}$$

(3) $$HCHE_{i}=\left\{\begin{array}{c} 0\;if\;T_{i}/(x_{i}-f\;\left(x\right)<z\\ \;\\ 1\;if\;T_{i}/(x_{i}-f\;\left(x\right)\geq z \end{array}\right\}$$

In equation (1), *A*_*k*_ is the average cost per visit to hospital or a doctor in 2 weeks, *B*_*k*_ is the average cost of drug purchases within a 2-week period, n is the number of visits to hospital or a doctor in a 2-week period, *C*_*k*_ is the average cost of hospitalization in 1 year, and m is the average hospitalization time in 1 year. In Equation 2, *a*_*k*_ is the average out-of-pocket health expenses per visit to hospital or a doctor in 2 weeks, *b*_*k*_ is the average out-of-pocket health expenses for medicines purchased over a 2-week period, n is the number of visits to hospital or a doctor in a 2-week period, *c*_*k*_ is the average out-of-pocket expenses for hospitalizations in 1 year, and m is the average hospitalization time in 1 year. In Equation 3, *HCHE*_*i*_ is HCHE in a given household *i, T*_*i*_ is annual total health expenditure of the household, *x*_*i*_ is the annual consumption expenditure of the household, *f(x)* is the annual food consumption expenditure of the household, and *z* is the threshold of 40% recommended by the WHO (32–34).

### Regression Analysis

Ordinary least square and binary multivariate logistic regression analyses of pooled cross-sectional data were carried out to evaluate the effects of health insurance and other factors on residents' THE, OOP, and HCHE. Variables for time and the interaction of time and social health insurance were also introduced to assess the impact of the universal health insurance system in China on residents' economic burden of disease.

## Results

### Characteristics of the Study Population

There were 25,042 survey participants from Jiangsu province including 10,931 urban residents (43.65%) and 14,111 rural residents (56.35%); 23.96% were from 2008, 37.07% were from 2013, and 38.97% were from 2018. The ratio of males to females was close to 1:1 (male:48.93, female:51.07%), and more than two-thirds of residents lived in a household of ≥2 members. The proportion of urban residents who had a higher education level was 76.81% which was larger than that of rural residents, while the latter had a higher proportion of individuals with the lowest education level (42.99%). The rates of employment in urban and rural areas were 49.46 and 79.27%, respectively, and there was a higher proportion of retirees in urban areas (32.77 vs. 2.57% in rural areas). Social health insurance coverage rate was >96% in both urban and rural areas. Details of other characteristics including depression, self-reported health status, chronic diseases, etc are shown in (Table 2).

**Table 2:** Characteristics of the study population according to area of residence (urban vs. rural).

	**Total**	**Urban**	**Rural**	***p***
	25,042	10,931 (43.65)	14,111 (56.35)	
Year				<0.001
2008	23.96	12.73	32.66	
2013	37.07	43.04	32.44	
2018	38.97	44.22	34.89	
**Predisposing factor**				
Sex				0.024
Male	48.93	48.12	49.56	
Female	51.07	51.88	50.44	
Age in years				<0.001
≤30	17.04	17.17	16.94	
30–45	22.76	21.08	24.07	
45–60	30.94	27.66	33.48	
>60	29.25	34.09	25.51	
Number of household members				<0.001
≤2	30.49	33.36	28.26	
>2	69.51	66.64	71.74	
Marital status				<0.001
Not married	17.78	18.82	16.97	
Married	82.22	81.18	83.03	
Education level				<0.001
Less than lower secondary	33.32	20.85	42.99	
Upper secondary/vocational training	53.28	55.96	51.20	
Tertiary	13.40	23.19	5.81	
Employment status				<0.001
Not employed	17.99	17.77	18.16	
Employed	66.26	49.46	79.27	
Retired	15.75	32.77	2.57	
**Enabling factor**				
Income				<0.001
Lowest income	25.01	9.01	37.40	
Lower income	25.55	20.57	29.40	
Higher income	25.00	31.71	19.80	
Highest income	24.45	38.71	13.40	
Social health insurance				0.008
No	3.50	3.85	3.23	
Yes	96.50	96.15	96.77	
Social health insurance type				<0.001
None	3.50	3.85	3.23	
UEMI	35.12	68.01	9.65	
URMI	11.28	23.68	1.68	
NCMS	28.55	2.47	48.76	
Other	21.54	1.99	36.68	
Financial subsidies				<0.001
No	96.24	97.15	95.54	
Yes	3.76	2.85	4.46	
**Need factors**				
Depression				<0.001
Not depressed	94.90	96.52	93.65	
Depressed	5.10	3.48	6.35	
Self-reported health status				<0.001
Poor (<50)	1.44	1.17	1.64	
Fair (50–70)	21.68	22.14	21.32	
Good (70–80)	27.53	30.14	25.51	
Excellent (80–100)	49.35	46.55	51.52	
Smoking				0.006
No	75.76	76.61	75.10	
Yes	24.24	23.39	24.90	
Drinking				0.005
No	76.77	77.61	76.11	
Yes	23.23	22.39	23.89	
Chronic diseases				<0.001
0	68.64	63.13	72.90	
1	22.54	25.86	19.97	
≥2	8.82	11.01	7.13	

*All data are shown as n (%) or %*.

*NCMS, New Rural Cooperative Medical Scheme; UEMI, Urban Employees' Medical Insurance; URMI, Urban Residents' Medical Insurance*.

*Lowest income, ≤¥11073.24; Lower income: ¥11073.24–20994.01; Higher income, ¥20994.01–34990.02; Highest income, >¥34990.02*.

### Social Health Insurance Coverage Rate and Economic Burden of Disease

**Figure 1:**
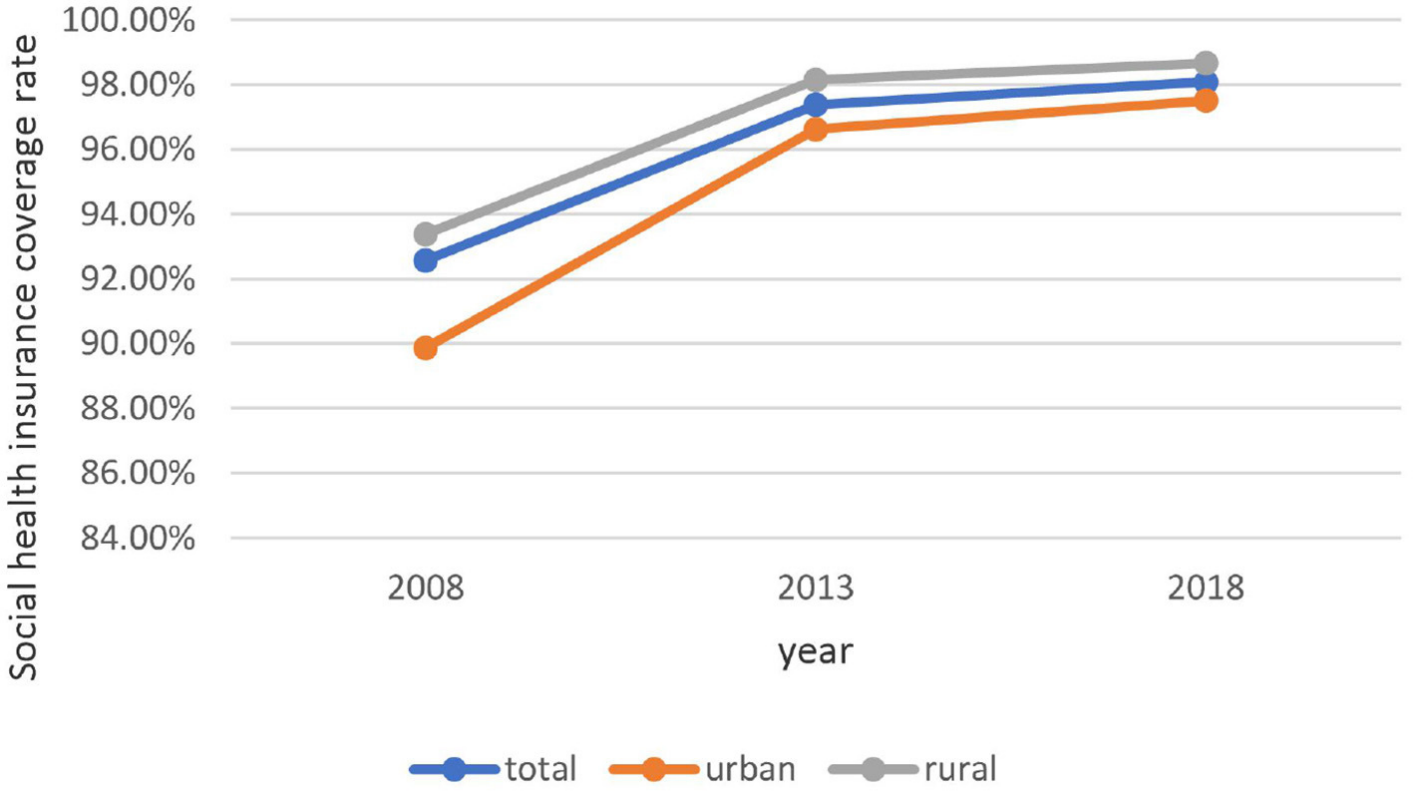
Trends in social health insurance coverage rate among residents of urban and rural areas from 2008 to 2018.

**Figure 2:**
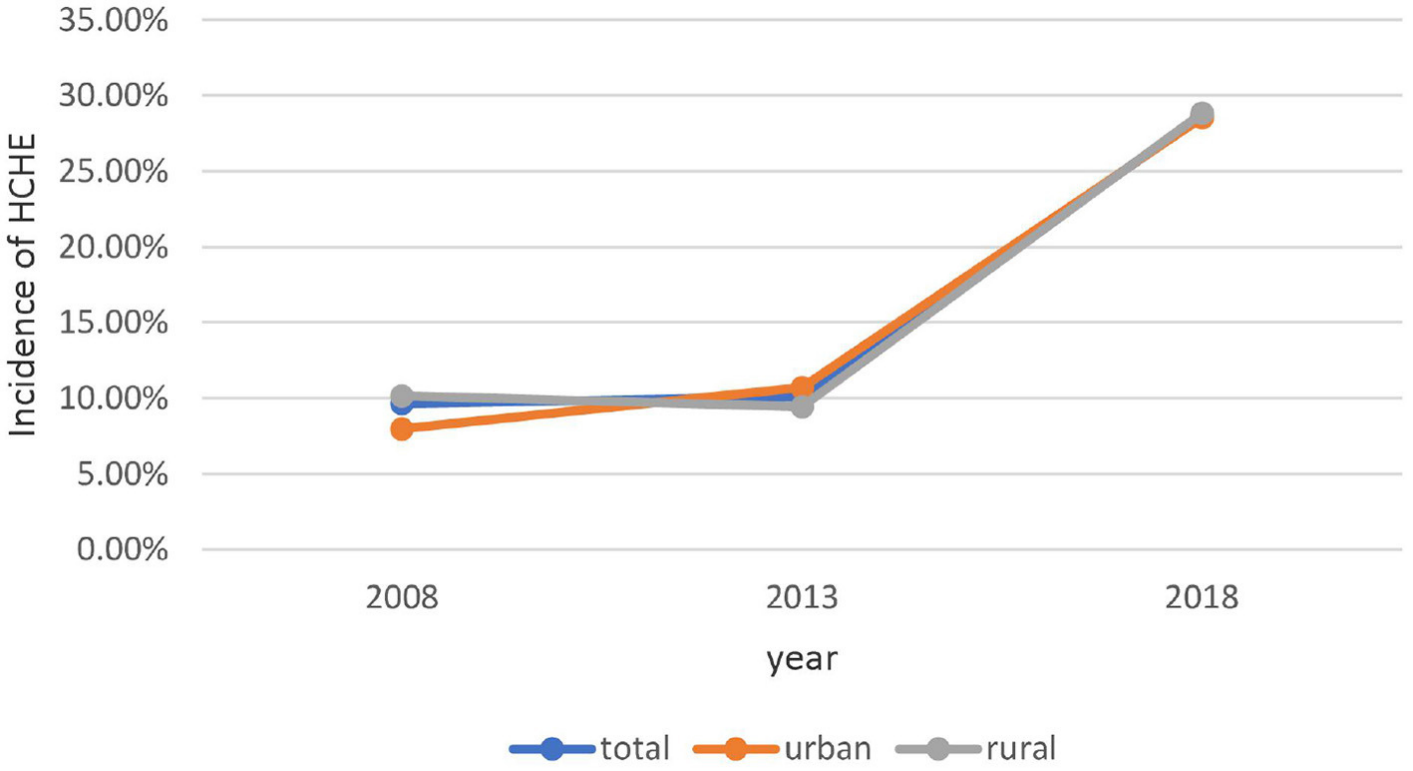
Trends in HCHE incidence among residents of urban and rural areas from 2008 to 2018.

**Figure 3:**
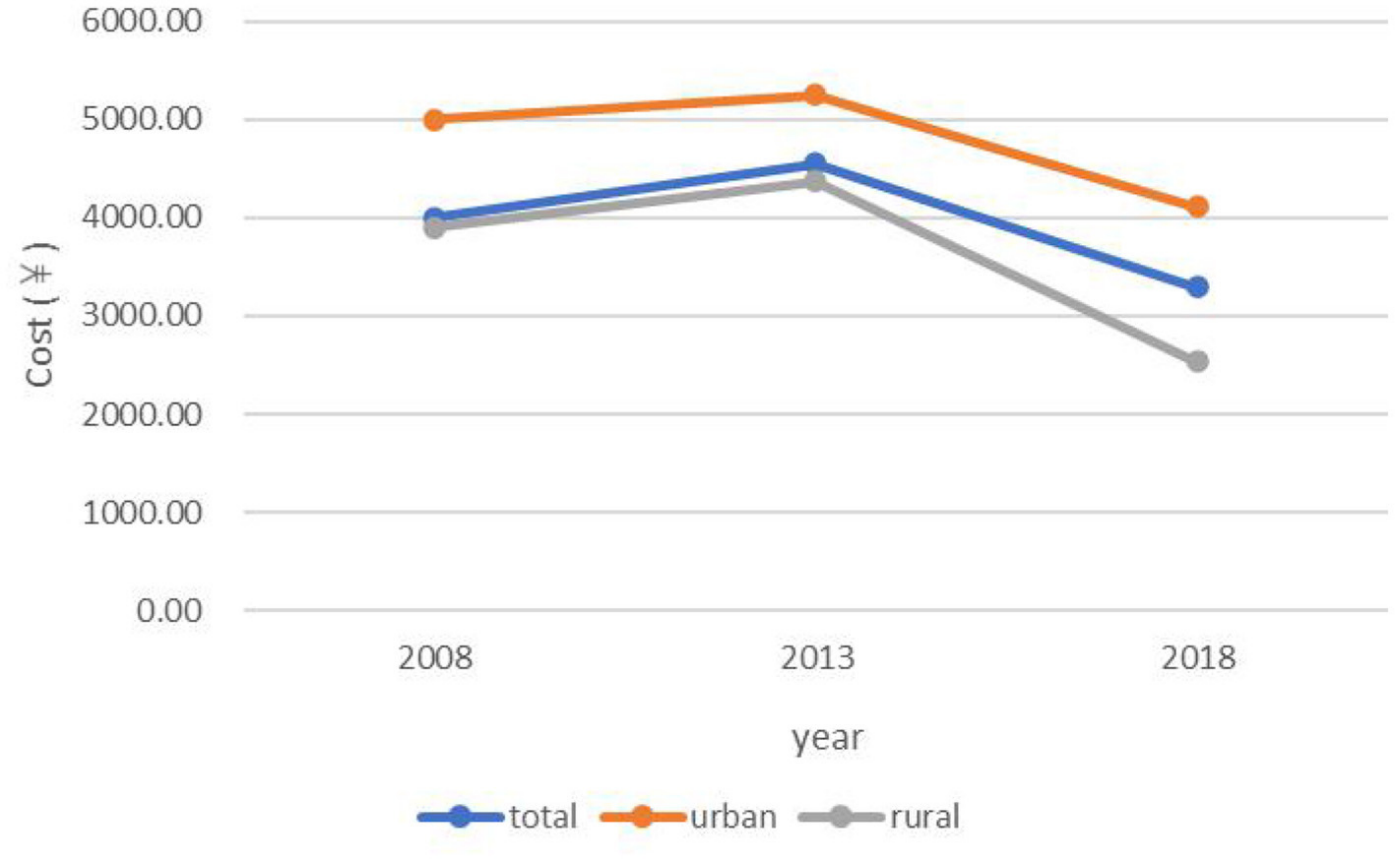
Trends in annual THE among residents of urban and rural areas from 2008 to 2018.

**Figure 4:**
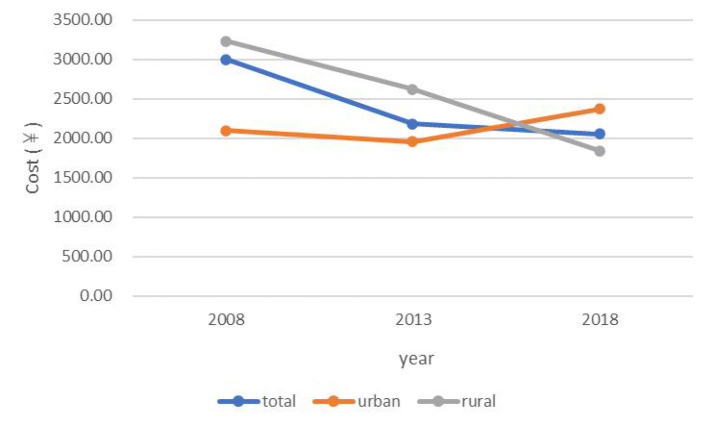
Trends in annual OOP among residents of urban and rural areas from 2008 to 2018.

Social health insurance coverage rate showed an increasing trend from 2008 to 2018 in the total population (2008: 92.57; 2013: 97.37; 2018: 98.08%) and among urban residents (2008: 89.87; 2013: 96.62; 2018: 97.50%) and rural residents (2008: 93.38; 2013: 98.14; 2018: 98.66%) (Figure 1). The incidence of HCHE was relatively stable from 2008 to 2013, and increased sharply from 2013 to 2018 in the total population (2013: 10.08; 2018: 28.67%) and in urban (2013: 10.71; 2018: 28.53%) and rural (2013: 9.44; 2018: 28.82%) areas (Figure 2). Annual THE showed an increasing trend before declining (2008: ¥4000; 2013: ¥4548.70; 2018: ¥3290.34) (Figure 3). THE was lower for rural residents (2008: ¥3900; 2013: ¥4373.75; 2018: ¥2534.19) than for urban residents (2008: ¥5000; 2013: ¥5248.50; 2018: ¥4112.92), and the degree of decline of THE between 2013 and 2018 was greater for the former. Annual OOP showed a downward trend for the total population (2008: ¥3000; 2013: ¥2186.88; 2018: ¥2056.46) and for rural residents (2008: ¥3235; 2013: ¥2624.25; 2018: ¥1840.53) but among urban residents, OOP initially decreased and then increased (2008: ¥2100; 2013: ¥1955.94; 2018: ¥2372.84) (Figure 4). Furthermore, OOP was lower for urban residents in 2008 and 2013 and higher in 2018 than for rural residents in those years.

### Multivariate Regression Analysis

#### Regression Analysis of THE

THE was significantly higher for insured residents than for uninsured residents in the total population (coef = 5.889) and in urban (coef = 7.325) and rural (coef = 5.190) areas (Table 3). THE was significantly higher in 2013 than in 2008 (total: coef = 5.852; urban: coef = 7.176; rural: coef = 5.468) but was significantly lower in 2018 (total: coef = −5.722; urban: coef = −7.330; rural: coef = −4.895). The coefficient of interaction of time (year) and social health insurance was significantly negative, and the absolute value of the coefficient was greater than that with health insurance in the total population (2013 × insurance: coef = −6.016; 2018 × insurance: coef = −6.247) and among urban (2013 × insurance: coef = −7.284; 2018 × insurance: coef = −7.644) and rural (2013 × insurance: coef = −5.578; 2018 × insurance: coef = −5.529) residents. The absolute values of the coefficients of the three variables were greater in the urban population than in the rural population.

**Table 3:** Regression analysis of annual total health expenditure (log) of urban and rural residents.

	**Total**		**Urban**		**Rural**	
	**coef**	**SE**	**coef**	**SE**	**coef**	**SE**
**Predisposing factor**						
Sex, ref: male	0.008	0.064	−0.104	0.091	0.084	0.089
**Age (years), ref:** **≤30**						
30–45	0.556^***^	0.107	0.240	0.149	0.809^***^	0.152
45–60	0.444^***^	0.109	0.288	0.162	0.671^***^	0.151
>60	0.630^***^	0.117	0.367^*^	0.175	0.891^***^	0.161
Number of household members, ref: ≤2	0.237^***^	0.055	0.124	0.079	0.306^***^	0.076
Marital status, ref: not married	0.618^***^	0.074	0.365^***^	0.103	0.792^***^	0.105
**Education level, ref: less than secondary**						
Upper secondary	0.348^***^	0.063	0.114	0.091	0.558^***^	0.086
Tertiary	0.608^***^	0.117	0.317^*^	0.143	0.943^***^	0.207
**Employment status, ref: unemployed**						
Employed	−0.029	0.071	−0.117	0.116	−0.054	0.093
Retired	−0.105	0.088	−0.119	0.108	0.197	0.186
**Enabling factor**						
**Income, ref:** **<**¥**11,663**						
¥11,663–21,869	0.178^*^	0.074	0.281^*^	0.141	0.168	0.091
¥21,869–36,911	0.213^**^	0.079	0.470^**^	0.139	0.089	0.104
>¥36,911	0.210^*^	0.086	0.363^*^	0.142	0.209	0.120
Social health insurance, ref: not insured	5.889^***^	0.175	7.325^***^	0.305	5.190^***^	0.222
Area of residence, ref: rural	0.414^***^	0.064				
Financial subsidies: ref: not receiving subsidies	0.233^*^	0.114	0.194	0.203	0.310^*^	0.140
**Need factor**						
Depression, ref: not depressed	0.599^***^	0.083	0.192	0.140	0.754^***^	0.104
**Self-reported health status, ref: poor**						
Fair	1.053^***^	0.126	0.693^***^	0.194	1.156^***^	0.166
Good	0.719^***^	0.133	0.319	0.200	0.860^***^	0.179
Excellent	0.783^***^	0.136	0.444^*^	0.205	0.882^***^	0.182
Smoking, ref: no smoking	−0.271^***^	0.075	−0.262^*^	0.113	−0.287^**^	0.099
Drinking, ref: no drinking	−0.219^**^	0.071	−0.202	0.105	−0.232^*^	0.096
**Chronic diseases, ref: 0**						
1	0.198^**^	0.064	−0.143	0.100	0.416^***^	0.084
≥2	0.538^***^	0.077	0.273^*^	0.113	0.677^***^	0.106
**Year, ref: 2008**						
2013	5.852^***^	0.376	7.176^***^	0.464	5.468^***^	0.652
2018	−5.722^***^	0.295	−7.330^***^	0.395	−4.893^***^	0.464
**Interaction of year and health insurance**						
YI2013	−6.016^***^	0.381	−7.284^***^	0.485	−5.578^***^	0.658
YI2018	−6.247^***^	0.300	−7.644^***^	0.414	−5.529^***^	0.469

* *p < 0.05*,

** *p < 0.01*,

*** *p < 0.001*.

*ref, reference; SD, standard deviation*.

THE of older residents was significantly higher than that of younger residents in the total population (30–45 years: coef = 0.556; 45–60 years; coef = 0.444; >60 years: coef = 0.630); however, in the urban population, only respondents over the age of 60 years had a higher THE. In the total population and among rural residents, THE was significantly higher in larger households (total: coef = 0.237; rural: coef = 0.306) and in respondents with a higher (upper secondary or tertiary) education level (total: coef = 0.348 and 0.608, respectively; rural: coef = 0.558 and 0.943, respectively) than in smaller households and respondents with a low education level. Compared to those who were unmarried, married respondents had a higher THE (total: coef = 0.618; urban: coef = 0.365; rural: coef = 0.792). THE was higher among urban residents than among residents of rural areas (coef = 0.414) and was higher among respondents who were receiving financial subsidies from the government (coef = 0.233). Respondents with depression (coef = 0.599); 1 or ≥2 chronic diseases (coef = 0.198 and 0.538, respectively); and fair, good, or excellent self-reported health status (coef = 1.053, 0.719, and 0.783, respectively) had significantly higher THE than those without depression or chronic diseases or who had poor self-rated health. Respondents who were smokers (coef = −0.271) or drank alcohol (coef = −0.219) had significantly lower THE than those who did not engage in these activities.

#### Regression Analysis of OOP

Social health insurance was associated with a significantly higher annual OOP (total: coef = 8.075; urban: coef = 8.024; rural: coef = 8.090) (Table 4). Among rural residents, OOP was significantly lower in 2013 and 2018 than in 2008 (2013: coef = −8.210; 2018: coef = −7.686), whereas among urban residents OOP was significantly higher in 2018 than in 2008 (2013: coef = −8.343; 2018: coef = 8.284). The coefficient of interaction of year and health insurance was significantly negative, and the absolute value was slightly higher than that with social health insurance.

**Table 4:** Regression of annual out-of pocket health expenditure of residents.

	**Total OOP (take the logarithm)**		**Urban OOP (take the logarithm)**		**Rural OOP (take the logarithm)**	
	**coef**	**SE**	**coef**	**SE**	**coef**	**SE**
**Predisposing factors**						
Sex, ref:male	−0.240^***^	0.062	−0.212^*^	0.087	−0.254^**^	0.089
**Age, ref:≤30**						
30–45	0.118	0.105	0.026	0.139	0.181	0.158
45–60	−0.202	0.108	0.018	0.152	−0.272	0.158
>60	−0.262^*^	0.117	−0.121	0.165	−0.276	0.169
Household numbers, ref: ≤2	0.126^*^	0.053	0.078	0.075	0.165^*^	0.075
Marital status, ref:not married	0.377^***^	0.072	0.205^*^	0.098	0.539^***^	0.105
**Education level, ref:less than secondary**						
Upper secondary	0.078	0.061	0.010	0.087	0.146	0.085
Tertiary	−0.033	0.111	−0.026	0.136	0.070	0.199
**Employment status, ref:unemployed**						
Employed	−0.148^*^	0.067	−0.181	0.108	−0.135	0.090
Retired	−0.121	0.082	−0.181	0.101	−0.044	0.181
**Enalbing factors**						
**Income, ref:≤**¥**11663**						
¥11663~21869	0.050	0.070	0.152	0.124	0.074	0.089
¥21869~36911	0.115	0.074	0.277^*^	0.123	0.054	0.098
>¥36911	0.124	0.080	0.188	0.127	0.200	0.114
Social health insurance, ref:not insured	8.075^***^	0.212	8.024^***^	0.326	8.090^***^	0.288
Area of residence, ref:rural	−0.227^***^	0.059				
Financial subsidies:ref:not receiving financial subsidies	−0.129	0.110	−0.094	0.192	−0.071	0.136
**Need factors**						
Depression, ref:not depressed	0.276^**^	0.080	0.047	0.132	0.389^***^	0.103
Self-reported health status, ref:poor						
Fair	−0.007	0.126	0.040	0.192	−0.062	0.168
Good	−0.436^**^	0.134	−0.387	0.199	−0.513^**^	0.182
Excellent	−0.441^**^	0.138	−0.330	0.204	−0.554^**^	0.187
Smoking,ref:no smoking	−0.259	0.072	−0.218^*^	0.108	−0.340^***^	0.097
Drinking,ref:no drinking	−0.241	0.067	−0.187	0.099	−0.260^**^	0.091
**Chronic diseases, ref:0**						
1	0.110	0.062	0.019	0.095	0.188^*^	0.083
≥2	0.383	0.073	0.301^**^	0.106	0.436^***^	0.101
**Year, ref:2008**						
2013	−8.172	0.352	−8.343^***^	0.438	−8.210^***^	0.605
2018	−7.924	0.288	8.284^***^	0.379	−7.686^***^	0.457
**Interaction of year and health insurance**						
YI2013	−8.378^***^	0.368	−8.452^***^	0.472	−8.410^***^	0.622
YI2018	−8.236^***^	0.305	−8.206^***^	0.414	−8.243^***^	0.477

* *p < 0.05*,

** *p < 0.01*,

*** *p < 0.001*.

*ref, reference; SD, standard deviation*.

Respondents over the age of 60 years and those who were employed had significantly lower OOP than those younger than 30 years of age or who were unemployed (age >60 years: coef = −0.262; employed: coef = −0.148). Respondents who were male, from a large household (>2), or who were married had a higher OOP than those who were female (coef = −0.240), from a small household (coef = 0.126), or who were unmarried (coef = 0.377). OOP was higher among urban residents in the second-highest income bracket than among those with the lowest income (coef 0.277), and was higher in rural residents and in the total population with depression than in respondents in these groups without depression (total: coef = 0.276; rural: coef =0.389). Respondents who reported their health status as “good” or “excellent,” were smokers, or who drank alcohol had a lower annual OOP than those who had poor self-reported health and did not smoke or drink. Additionally, OOP was higher for respondents with chronic diseases (≥2) than for those without chronic diseases (urban: coef = 0.301; rural: coef = 0.436).

#### Logistic Regression Analysis of HCHE

The associations between social health insurance and other socioeconomic factors and HCHE are summarized in (Table 5). The probability of HCHE was significantly lower in households with insured residents (total: OR = 0.573; urban: OR = 0.597; rural: OR = 0.580). Compared to 2008, the probability of HCHE was increased in 2018 (total: OR = 3.118; urban: OR = 4.495; rural: OR = 2.614), and the OR of the interaction of the year 2018 and health insurance was significant in the total population (OR = 1.784), which was contrary to the effect of social health insurance on HCHE. By comparing the ORs of the two variables, we found that the positive interaction effect of the year 2018 and insurance on HCHE was greater than the negative effect of health insurance on HCHE.

**Table 5:** Logistic regression analysis of the likelihood of household catastrophic health expenditure of urban and rural residents.

	**Total**		**Urban**		**Rural**	
	**OR**	**SE**	**OR**	**SE**	**OR**	**SE**
**Predisposing factor**						
Sex, ref: male	0.786^***^	0.036	0.772^***^	0.052	0.782^***^	0.050
**Age (years), ref:** **≤30**						
30–45	0.791^**^	0.057	0.857	0.089	0.750^**^	0.075
45–60	0.685^***^	0.051	0.709^**^	0.081	0.638^***^	0.066
>60	0.891	0.072	0.827	0.103	0.895	0.097
Number of household members, ref: ≤ 2	0.574^***^	0.023	0.688^***^	0.041	0.496^***^	0.028
Marital status, ref: not married	1.190^**^	0.064	1.254^**^	0.098	1.126	0.084
**Education level, ref: less than secondary**						
Upper secondary	0.910^*^	0.043	1.024	0.073	0.839^**^	0.055
Tertiary	0.868	0.070	0.839	0.088	1.085	0.145
**Employment status, ref: unemployed**						
Employed	0.909	0.048	0.825^*^	0.070	0.980	0.067
Retired	1.088	0.073	1.139	0.098	1.234	0.186
**Enabling factor**						
**Income, ref:** **<**¥**11,663**						
¥11,663–21,869	0.575^***^	0.030	0.598^***^	0.057	0.603^***^	0.039
¥21,869–36,911	0.385^***^	0.022	0.428^***^	0.040	0.347^***^	0.028
>¥36,911	0.227^***^	0.015	0.222^***^	0.022	0.270^***^	0.026
Social health insurance, ref: not insured	0.573^***^	0.066	0.597^*^	0.129	0.580^***^	0.083
Area of residence, ref: rural	0.344^***^	0.062				
Financial subsidies: ref: not receiving subsidies	1.781^***^	0.145	1.943^***^	0.275	1.712^***^	0.171
**Need factor**						
Depression, ref: not depressed	1.291^***^	0.093	1.016	0.132	1.432^***^	0.125
**Self-reported health status, ref: poor**						
Fair	0.618^***^	0.063	0.498^***^	0.085	0.739^*^	0.097
Good	0.403^***^	0.042	0.353^***^	0.061	0.448^***^	0.061
Excellent	0.310^***^	0.032	0.265^***^	0.046	0.360^***^	0.048
Smoking, ref: no smoking	0.884^*^	0.047	0.809^**^	0.066	0.924	0.065
Drinking, ref: no drinking	0.779^***^	0.040	0.799^**^	0.062	0.763^***^	0.054
**Chronic diseases, ref: 0**						
1	1.355^***^	0.064	1.242^**^	0.088	1.473^***^	0.094
≥2	1.782^***^	0.111	1.854^***^	0.163	1.699^***^	0.153
**Year, ref: 2008**						
2013	1.147	0.265	1.737	0.510	0.645	0.320
2018	3.188^***^	0.659	4.495^***^	1.256	2.614^**^	0.917
**Interaction of year and health insurance**						
YI2013	1.241	0.294	1.065	0.336	2.006	1.003
YI2018	1.784^**^	0.376	1.691	0.503	1.994	0.705

* *p < 0.05*,

** *p < 0.01*,

*** *p < 0.001*.

*ref, reference; SD, standard deviation*.

The probability of HCHE was significantly lower in households with females (total: OR = 0.786; urban: OR = 0.772; rural: OR = 0.782) (Table 5). Households with residents aged 45–60 years also had a significantly lower probability of HCHE (OR = 0.685). Having fewer household members (≤2) was significantly and positively associated with HCHE compared to living in a household with more members (>2) (total: OR = 0.574; urban: OR = 0.688; rural: OR = 0.496). The probability of HCHE was higher for households in the total population and in urban areas with married respondents (total: OR = 1.190; urban: OR = 1.254), and lower for those with respondents with a high education level (total: OR = 0.910; rural: OR = 0.839). Furthermore, the probability of HCHE was lower for households in urban areas with employed respondents (OR = 0.825). All of the enabling and need factors had a significant impact on the probability of HCHE. Households in which respondents had a higher income or better health, smoke or drank alcohol had a significantly lower incidence of HCHE. Households in rural areas (OR = 0.344), receiving government financial assistance (OR = 1.781), or with respondents who experienced depression (OR = 1.291) or had 1 or ≥2 chronic diseases (OR = 1.355 and 1.782, respectively) had a significantly higher probability of HCHE.

## Discussion

China established a universal social health insurance system in 2003 to ensure the health of the population through shared economic risks of disease and improved accessibility of health services (35–37). However, whether universal social health insurance coverage has alleviated the economic burden of disease for individuals has not been systematically investigated. To this end, the present study analyzed the trends in residents' economic burden of disease (including THE, OOP, and HCHE) from 2008 to 2018 and how this was affected by universal social health insurance coverage and other socioeconomic factors using NHSS data from Jiangsu province.

## Trends in Social Health Insurance Coverage Rate and Economic Burden of Disease

Social health insurance coverage has gradually increased in China; in 2018, the coverage rate in urban and rural areas exceeded 97%, which was close to full coverage for the population. Additionally, the health insurance coverage rate was consistently higher in rural areas than in urban areas, which may be related to differences in policy implementation.

Consistent with previous studies (38–40), THE was significantly higher for urban residents than for rural residents. This may be attributable to the higher degree of socioeconomic development and better medical resource allocation in urban areas that make health services more affordable and accessible, thereby increasing health service expenditure. Also in agreement with earlier findings (38, 41) was our observation that OOP was higher for rural residents than for urban residents, but only in the early part of the survey period; that is, the gap between the two groups decreased as social health insurance coverage became widespread. The incidence of HCHE was also higher among rural residents, which was likely due to their lower income level and limited medical security level (4). Therefore, the government should improve allocation of health resources between urban and rural areas and optimize health insurance systems in the latter by increasing financial support and income levels to reduce the economic burden and risks of disease.

From 2008 to 2018, THE increased before decreasing. The first part of this trend may be related to improvements in the medical system and health insurance coverage after the medical reforms of 2009, which increased utilization of health services and THE (42). During implementation of the reforms, the regulations of the medical insurance system (e.g., cancellation of drug bonuses) were continuously updated; this along with the decline of drug prices and moral hazard of social health insurance resulted in a decrease in THE. In the total population and among rural residents, OOP decreased from 2013 to 2018 whereas in urban areas it increased after 2013, exceeding the OOP of rural residents in 2018. The high THE among urban residents may have led to a higher OOP even after reimbursement. Establishing critical illness insurance can potentially reduce OOP in this group. The incidence of HCHE remained relatively stable between 2008 and 2013 and then rose sharply from 2013 to 2018, which may be related to advances in medical technology, the aging of the population, and changes in disease burden.

## Effect of Health Insurance and Universal Coverage on the Economic Burden of Disease

Having health insurance increased residents' THE, which contradicts the findings of some previous surveys (9, 21, 43). This may be because social medical insurance has reduced the unmet demand for health services. Moral hazards of suppliers caused by information asymmetry in health services [“pushing up prices” (44), “inducing demand,” or “overtreatment”] may have also contributed to the increase in THE (28). Hence, the government should strengthen the monitoring of health insurance funds and improve related regulations to promote the effective utilization of health services while reducing moral hazards in order to control THE (19). Although having social health insurance significantly increased THE in the population, universal social health insurance coverage had the opposite effect. The coefficient of the interaction of health insurance and time was significantly negative and the absolute value was greater than that of the coefficient of social health insurance, implying that universal coverage and improvements in the health insurance system in China can mitigate the excessive increase in THE.

In accordance with published reports (3, 16, 19, 44), respondents in urban and rural areas with health insurance had significantly higher OOP than those without insurance. Social health insurance has eased the demand for health services, thereby promoting their utilization. Additionally, rapid economic development and changes in the disease spectrum have increased medical costs including OOP for residents. However, the positive effect of having medical insurance on OOP was diminished by universal social health insurance coverage, which was similar to Finkelstein & McKnight's finding and Nguyen H's report (45, 46), possibly because it has gradually improved the level of reimbursement. Thus, although having social health insurance increased OOP, the net effect of universal social health insurance coverage on OOP was slightly negative and alleviated to some extent the economic burden of disease.

The probability of HCHE was significantly lower among residents with health insurance, implying that having insurance protected residents from the economic risks of disease as demonstrated in other surveys (4, 5, 15, 16). However, with economic development and rising medical costs, the incidence of HCHE has increased significantly, as evidenced by a coefficient >1 for the interaction between the year 2018 and social health insurance. This indicates that although having health insurance reduced the probability of HCHE, this effect is gradually diminishing, such that the aggregate effect of universal of social health insurance coverage actually increased rather than decreased the probability of HCHE. That is, as the increase in medical costs has exceeded that of reimbursement, social health insurance is no longer sufficient to protect people from the economic risks of disease. To overcome this problem, the government should increase financial subsidies, modify medical insurance catalogs, and expand the scope of reimbursement for diseases (4). Additionally, a multilevel medical security system that includes medical assistance and critical illness insurance can potentially reduce the risk of HCHE.

## Other Socioeconomic Factors Associated With the Economic Burden of Disease

THE was significantly higher for respondents over the age of 60 years, which is consist with previous findings (47, 48). This may be because the elderly are more likely to suffer from diseases than younger people due to a decline in physical health. The effect was more pronounced in rural areas, possibly reflecting their inferior living conditions compared to urban residents. However, OOP was significantly lower for respondents older than 60 years than for those aged ≤30 years, which differs from the results of another study (49). This discrepancy may be attributable to special medical insurance policies for the elderly such as reimbursements for chronic or geriatric diseases.

As reported in earlier studies (48, 50, 51), respondents with higher education and income levels had a significantly higher THE but lower risk of HCHE. This may be because people with higher education and income are more likely to take the initiative to seek medical treatment in a timely manner and thereby prevent critical diseases and the associated economic burden. OOP and HCHE were higher among rural residents than in the urban population, highlighting the disparities in economic development and medical insurance policies between urban and rural areas. Additionally, OOP and incidence of HCHE were higher for respondents with poor self-reported health and chronic diseases (4, 52, 53) due to the greater demand for and utilization of health services by these individuals. Therefore, the government should pay more attention to vulnerable groups such as the elderly, rural residents, and people with a low level of education and chronic diseases (4) to avoid “poverty due to illness” in China. As for the “drinking” and “smoking”, the results showed that households in which smokers and drinkers were more likely to occur catastrophic health expenditure. However, since the analysis was not the regression of panel data, it was impossible to infer causality, which could only reflect the correlation between the two. Therefore, the possible reason may be that the members of families with catastrophic health expenditure were not allowed to smoke and drink due to their physical conditions and economic conditions, which showed that the probability of HCHE in families with smokers and drinkers was lower.

## Strengths and Limitations

The paper is the first article to explore the policy effect of universal health insurance coverage process on Residents' disease economic burden in China by using data spanning 10 years (2008, 2013, and 2018). Additionally, Our sample is based on the whole population which will be more universal. Nevertheless, there are still some limitations. Firstly, it was not realistic to include all possible impact factors in our regression models, which may have led to oversimplification in our conclusions. Secondly, we used NHSS data from Jiangsu province, which may not be representative of the whole country. Finally, given that we used pooled cross-sectional data, we were unable to deduce causality.

## Conclusion

Social health insurance had a significant positive effect on the annual THE and OOP and a significant negative effect on HCHE in urban and rural residents of Jiangsu province, China. However, universal health insurance coverage could alleviated the economic burden of disease on individuals (THE and OOP) while it was insufficient to protect against the economic risk of diseases (HCHE). There are still discrepancies between urban and rural areas in terms of the impact of health insurance; moreover, the economic burden of disease is greater for people living in rural areas or who are elderly, less educated, have a lower income, are in poor health, or have chronic diseases. Therefore, the government should shift the emphasis of social health insurance from quantity to quality and take multiple measures to reduce the economic risks of disease—especially in these vulnerable groups—in order to promote the development of the economy and society.

## Data Availability Statement

The original contributions presented in the study are included in the article/supplementary material, further inquiries can be directed to the corresponding author.

## Ethics Statement

This study was approved by the Academic Research Ethics Committee of Nanjing Medical University. All procedures were in accordance with the ethical standards of the Helsinki Declaration. Participants provided informed consent prior to data collection.

## Author Contributions

SZ, CZ, QY, and ZW: conceptualization, methodology, and software. SZ, QY, and ZW: validation, formal analysis, investigation, and writing—review and editing. ZW: data curation, supervision, and project administration. SZ and CZ: writing—original draft preparation. All authors have read and agreed to the published version of the manuscript.

## Funding

This research was funded by the Excellent Innovation Team of the Philosophy and Social Sciences in the Universities and Colleges of Jiangsu Province.

## Conflict of Interest

The authors declare that the research was conducted in the absence of any commercial or financial relationships that could be construed as a potential conflict of interest.

## Publisher's Note

All claims expressed in this article are solely those of the authors and do not necessarily represent those of their affiliated organizations, or those of the publisher, the editors and the reviewers. Any product that may be evaluated in this article, or claim that may be made by its manufacturer, is not guaranteed or endorsed by the publisher.
